# Simvastatin Prevents Liver Microthrombosis and Sepsis Induced Coagulopathy in a Rat Model of Endotoxemia

**DOI:** 10.3390/cells11071148

**Published:** 2022-03-29

**Authors:** Vincenzo La Mura, Nicoletta Gagliano, Francesca Arnaboldi, Patrizia Sartori, Patrizia Procacci, Luca Denti, Eleonora Liguori, Niccolò Bitto, Giuseppe Ristagno, Roberto Latini, Daniele Dondossola, Francesco Salerno, Armando Tripodi, Massimo Colombo, Flora Peyvandi

**Affiliations:** 1 Fondazione I.R.C.C.S. Ca’ Granda, Ospedale Maggiore Policlinico, U.O.C. Medicina Generale Emostasi e Trombosi, 20122 Milan, Italy; niccolo.bitto@policlinico.mi.it (N.B.); armando.tripodi@unimi.it (A.T.); flora.peyvandi@unimi.it (F.P.); 2 CRC “A.M. e A. Migliavacca” per lo Studio e la Cura delle Malattie del Fegato, Università degli Studi di Milano, 20122 Milan, Italy; 3 Dipartimento di Fisiopatologia dei Trapianti, Università degli Studi di Milano, 20132 Milan, Italy; eleonora.liguori1982@gmail.com (E.L.); giuseppe.ristagno@unimi.it (G.R.); dondossola.daniele@gmail.com (D.D.); 4 Dipartimento di Scienze Biomediche per la Salute, Università degli Studi di Milano, 20133 Milan, Italy; nicoletta.gagliano@unimi.it (N.G.); francesca.arnaboldi1@unimi.it (F.A.); patrizia.sartori@unimi.it (P.S.); patrizia.procacci@unimi.it (P.P.); luca.denti1@studenti.unimi.it (L.D.); francesco.salerno@unimi.it (F.S.); 5 Fondazione I.R.C.C.S. Ca’ Granda, Ospedale Maggiore Policlinico, U.O.C. Anestesia e Rianimazione, 20122 Milan, Italy; 6 Dipartimento di Ricerca Cardiovascolare, Istituto di Ricerche Farmacologiche Mario Negri I.R.C.C.S., 20156 Milan, Italy; roberto.latini@marionegri.it; 7 U.O. Chirurgia Generale e dei Trapianti di Fegato, Fondazione IRCCS Ca′ Granda Ospedale Maggiore Policlinico, 20122 Milan, Italy; 8 Liver Center IRCCS San Raffaele Hospital, 20132 Milan, Italy; mcolombo46@yahoo.it

**Keywords:** sepsis, sinusoidal endothelial cells, thrombomodulin, coagulation

## Abstract

Background: Endotoxemia causes endothelial dysfunction and microthrombosis, which are pathogenic mechanisms of coagulopathy and organ failure during sepsis. Simvastatin has potential anti-thrombotic effects on liver endothelial cells. We investigated the hemostatic changes induced by lipopolysaccharide (LPS) and explored the protective effects of simvastatin against liver vascular microthrombosis. Methods and results: We compared male Wistar rats exposed to LPS (5 mg/kg one i.p. dose) or saline in two experimental protocols—placebo (vehicle) and simvastatin (25 mg/kg die, orally, for 3 days before LPS). Morphological studies were performed by light- and electron-microscopy analyses to show intravascular fibrin deposition, vascular endothelial structure and liver damage. Peripheral- and organ-hemostatic profiles were analyzed using whole blood viscoelastometry by ROTEM, liver biopsy and western-blot/immunohistochemistry of thrombomodulin (TM), as well as immunohistochemistry of the von Willebrand factor (VWF). LPS-induced fibrin deposition and liver vascular microthrombosis were combined with a loss of sinusoidal endothelial TM expression and VWF-release. These changes were associated with parenchymal eosinophilia and necrosis. ROTEM analyses displayed hypo-coagulability in the peripheral blood that correlated with the degree of intrahepatic fibrin deposition (*p* < 0.05). Simvastatin prevented LPS-induced fibrin deposition by preserving TM expression in sinusoidal cells and completely reverted the peripheral hypo-coagulability caused by endotoxemia. These changes were associated with a significant reduction of liver cell necrosis without any effect on eosinophilia. Conclusions: Simvastatin preserves the antithrombotic properties of sinusoidal endothelial cells disrupted by LPS, deserving pharmacological properties to contrast sepsis-associated coagulopathy and hepatic failure elicited by endotoxemia

## 1. Introduction

Sepsis is the body’s extreme response to infections and represents a global health priority [1]. It accounts for up to 30% of in-hospital mortality as a consequence of a dysregulated host immune response, which ultimately causes multiple organ dysfunction and death [2]. The liver is one of the target organs during sepsis. Indeed, more than one-third of patients develop hepatic dysfunction, as heralded by progressive hyperbilirubinemia [3]. Proper liver failure has been described in up to 22% of cases, which confirms the prognostic role played by the liver in this setting [4]. Blood coagulation disorders are invariably associated with severe infections, represent a determinant pathogenic mechanism of organ failure and can predict sepsis-related mortality [5,6]. These hemostatic changes characterize the so-called sepsis-associated coagulopathy, which correlates with intravascular microthrombosis, a driver of tissue ischemia and organ failure [7]. This notwithstanding, the use of anticoagulants in sepsis has been questioned. Indeed, results have consistently demonstrated that the positive pathogenic effect on organ microthrombosis is counterbalanced by the intrinsic higher hemorrhagic risk posed by the treatment [8]. Therefore, only a selected group of patients could take advantage of this pharmacological approach, and new strategies of treatment remain an urgent need for patient care.

Vascular endothelial dysfunction (ED) represents an ideal alternative target of therapy in sepsis [2] since it precedes the development of organ dysfunction and participates in the pathogenesis of organ perfusion, vascular permeability and activation of the coagulation cascade [3]. Studies on animal models of cirrhosis, endotoxemia and ischemia-reperfusion injury have demonstrated that simvastatin protects liver sinusoidal endothelial cells by the development of ED, as defined by the impairment of the nitric oxide (NO)-dependent vasodilatory function of liver sinusoids [9,10,11,12]. These studies, mainly focusing on the vasomotor function of the endothelium, have been promoted to phase II randomized controlled trials, demonstrating that simvastatin can reduce portal pressure and deserves a potential impact on mortality in cirrhosis [13,14,15]. Importantly, the ability of simvastatin to ameliorate the vasomotor function of sinusoidal endothelial cells parallels the efficacy to maintain the expression of thrombomodulin (TM), which is one of the hallmarks of a preserved antithrombotic function of the endothelium in microcirculation [11]. Therefore, it has been argued that simvastatin possesses vascular anticoagulant properties that may preserve the liver from microthrombosis [16]. 

Our study aimed to investigate the intrahepatic and peripheral changes of coagulation induced by lipopolysaccharide (LPS), a bacterially derived by-product sustaining endotoxemia during gram-negative bacterial infections. We also tested the efficacy of simvastatin to prevent these changes and necrosis of the liver in association with the maintenance of TM expression in sinusoidal endothelial cells. 

## 2. Materials and Methods

### 2.1. Animals and Ethical Statements

Male Wistar rats (body weight: 275–300 g) were caged in pairs in a 12/12 h light–dark cycle, temperature- and humidity-controlled environment, with free access to food and water in a Specific pathogen-free laboratory at the Istituto di Ricerche Farmacologiche Mario Negri IRCCS, Milan, Italy. All the animals received human care and all the study protocols were planned in agreement with the Guide for the Care and Use of Laboratory Animals (National Institutes of Health, 8th edition, 2011) and conducted after approval by the Animal Care of the Istituto di Ricerche Farmacologiche Mario Negri and the Italian Ministry of Health. The number and morphometry of rats included in each experimental condition are detailed in Supplementary Tables S1–S3 and Supplementary Figure S1.

### 2.2. Experimental Groups

Experiments were conducted during light cycle. The main experimental protocol consisted of comparing the effects of LPS (5 mg/kg) vs. saline 24 h after the intraperitoneal injection in two experimental conditions: placebo (vehicle) and three-day pre-treatment with simvastatin (25 mg/kg of body weight, orally), as previously described [10]. Additional experiments were also planned to explore the effects of LPS (5 mg/kg) at 3–6 to 12–24 h after intraperitoneal injection (time-dependent effect of LPS) and the effects of different doses of LPS at 1–3–5 mg/kg at 24 h (dose-effect of LPS). All rats were randomly assigned to different treatments as a single animal unit. Experimental groups are also displayed in Supplementary Figure S1.

### 2.3. Sample Collection

Twenty-four hours after LPS or saline injection rats received general anesthesia (Ketamine i.p. route at 75 mg/kg) and analgesia (Medetomidine i.p. route at 0.5 mg/kg) and subsequent laparotomy, blood samples from the inferior vena cava were collected for ROTEM analyses. The animals were then sacrificed and their liver fragments collected for morphological and molecular analysis. Liver fragments for morphology were washed in 0.1 M phosphate-buffered saline (PBS), pH 7.4, and immediately processed for light and electron microscope analysis or immersed in liquid nitrogen for molecular evaluation. 

### 2.4. Antibodies and Reagents

Antibodies used for immunohistochemistry, immunofluorescence and Western blot analysis are listed in Supplementary Material.

### 2.5. Coagulation, Endothelium and Liver

The intrahepatic effect of LPS on coagulation was measured by the levels of fibrin deposition inside the sinusoids as marker of microthrombosis [17]. The systemic effect of LPS on the coagulation was analyzed with rotational thromboelastometry (ROTEM, gamma equipment). TM expression on liver sinusoidal endothelial cell surface was considered marker of preserved antithrombotic phenotype [17,18]. Von Willebrand factor was used to evidence the participation of the endothelium in the thrombus generation [19,20].

### 2.6. Peripheral Blood Analysis (ROTEM)

Citrated whole blood samples from inferior vena cava were analyzed with ROTEM, gamma equipment within 120 min after venipuncture, according to the manufacturer’s instructions. Briefly, blood was recalcified and coagulation was initiated by rabbit-derived tissue factor (EXTEM). The following parameters were recorded: the clotting time (CT), which is the time from the initiation of the measurement to the formation of a 2 mm clot amplitude; the clot formation time (CFT), which is the time needed to reach the clot amplitude from 2 to 20 mm; the maximum clot firmness (MCF), which is the maximum clot amplitude; and the maximum clot firmness time (MCF-T), which is the time needed to reach the MCF.

### 2.7. Histological Analysis

Liver fragments were fixed in 10% formalin in PBS, dehydrated, paraffin-embedded and serially cut. Five μm thick sections were stained with hematoxylin-eosin to evaluate liver structure or incubated with specific antibodies for immunohistochemistry and immunofluorescence analyses. To assess liver structure, hematoxylin–eosin-stained sections for each liver sample were analyzed in blind under a light microscope (Nikon Eclipse E600, Nikon, Tokyo, Japan) by three operators (NG, FA, LC), using a semiquantitative four-point scoring system. Specifically, hepatocytes with acidophilic bodies and liver cell necrosis were graded as follows: absence, + mild, ++ moderate, +++ severe. 

### 2.8. Immunohistochemistry and Image Analysis

Fibrin deposition and the expression of TM and VWF were analysed by immunohistochemistry. Fibrin deposition was assessed by an anti-fibrinogen antibody, as previously reported [21]. After deparaffinization and rehydration of liver sections, endogenous peroxidase activity was quenched by 3% H_2_O_2_ in PBS for 20 min at 37 °C and antigen retrieval was performed by incubating the sections with proteinase K in Tris-EDTA buffer (TE), pH8, for 20 min at 37 °C. Sections were incubated with primary antibodies anti-fibrinogen (dilution 1:400 in PBST), anti-TM (1:20 in PBST) and anti- VWF (1:500 in PBST) for 1 h at room temperature. After incubation with HRP-conjugated secondary antibody for 1 h at room temperature (dilution 1:400 in PBST), chromogenic 3,3′-diaminobenzidine (DAB) substrate was added to visualize the expression of the target proteins and sections were then counterstained with hematoxylin. Negative controls were obtained, omitting the primary antibody. The sections were observed under a light microscope (Nikon Eclipse E600, Nikon, Tokyo, Japan) and photographed by a digital camera. Fibrin deposition was assessed by (blind) triplicate observations by image analysis, quantifying the immunoreactive area for fibrin, expressed as % relative to the total area of the section (at least 8 fields).

### 2.9. Immunofluorescence

The expression of TM in endothelial cells lining liver sinusoids was assessed by immunofluorescence analysis. Liver sections were incubated with anti-TM antibody (1:40 in PBST) for 1 h at room temperature and with an anti-sheep Alexa488-conjugated secondary antibody (1:500 in PBST) for 1 h. Nuclei were stained with DAPI. To demonstrate the co-localization of fibrin and VWF, double labelling of paraffin-embedded sections of a human blood clot was performed and revealed by secondary antibodies conjugated with Alexa594 and Alexa488, respectively. Sections were observed by a WD THUNDER Imager Tissue 3D (Leica Microsystems, Buccinasco-Milan, Italy).

### 2.10. Transmission Electron Microscopy

Liver fragments obtained from the same lobe of each animal used for light microscopy were quickly fixed in 2% paraformaldehyde and 2% glutaraldehyde in 0.1 M sodium cacodylate buffer (pH 7.3). After fixation, samples were washed in cacodylate buffer and postfixed at 0 °C for 1.5 h in 2% osmium tetroxide in the same buffer. The specimens were subsequently washed in distilled water, stained en block in 2% aqueous uranyl acetate, dehydrated through an ascending series of ethanol, embedded in Epon–Araldite resin and oriented for the classic lobule cross-sectioning. From each sample, 0.5 μm semithin sections were obtained with an LKB III ultramicrotome, stained with 0.5% toluidine blue in 1% sodium borate and examined by light microscope (Carl Zeiss Axiophot Photomicroscope, Oberkochen, Germany) to check the quality of fixation. Ultra-thin sections (50–70 nm thick), cut by a Leica Supernova ultra-microtome, were mounted on formvar-coated copper grids. Sections were stained with lead citrate and examined under the transmission electron microscope Zeiss EM10 (Carl Zeiss, Oberkochen, Germany).

### 2.11. Real-Time PCR

Liver homogenates were prepared using a Tissue Lyzer (Qiagen, Milan, Italy). Total RNA was isolated by Tri-Reagent (Sigma-Aldrich, Milan, Italy) and 1µg of total RNA was reverse-transcribed in 20 µL final volume of reaction mix (BioRad Segrate-Milan, Italy). mRNA levels for KLF-2 were assessed and normalized on the housekeeping 18 s used as endogenous control. The primers were as follows: KLF-2: sense ACTTGCAGCTACACCAACTG, antisense CTGTGACCCGTGTGCTTG; 18 s sense CTGCCCTATCAACTTTCGATGGTAG, antisense CCGTTTCTCAGGCTCCCTCTC. Amplification reactions were conducted in triplicate in a 96-well plate in a final volume of 20 µL per well, containing 10 µL of 1× SYBR Green Supermix (BioRad, Segrate-Milan, Italy), 2 µL of template and 300 pmol of each primer in a Bioer LineGene 9600 (Bioer, Hangzhou, China). The cycle threshold (Ct) was determined and gene expression levels relative to that of 18 s were calculated by the 2^−ΔΔCt^ method.

### 2.12. Western Blot 

Liver homogenates were prepared in Tris-HCl 50 mM pH 7.6, 150 mM NaCl, 1% Triton X-100, 5 mM EDTA, 1% SDS, proteases inhibitors and 1 mM sodium orthovanadate using a Tissue Lyzer (Qiagen, Italy), and centrifuged at 14,000× *g*, for 10 min at 4 °C to remove cell debris. For analysis, liver homogenates (50 µg of total proteins) were diluted in SDS-sample buffer, loaded on 10% SDS-polyacrylamide gel, separated under reducing and denaturing conditions at 80 V, and electro-blotted to a nitrocellulose membrane in 0.025 M Tris, 192 mM glycine, 20% methanol and pH 8.3. Membranes were blocked for 1 h in 5% skimmed milk in TBST and incubated with the antibodies anti-TM (1:2000) for 1 h at room temperature. After washing, membranes were incubated with HRP-conjugated secondary antibodies (1:6000 in TBST). To confirm equal loading, membranes were re-probed by monoclonal antibody to actin (1:7500 in TBST). Immunoreactive bands were revealed by the Opti-4CN substrate (Bio Rad, Segrate-Milan, Italy) and quantified by densitometric scanning. 

### 2.13. Statistical Analysis

SPSS 26.0 statistical package (IBM) was used for data analysis. Groups were compared for continuous variables by the U-Mann Whitney test or the Jonckheere–Terpstra as the most opportune non-parametric test. Pearson coefficient resumed correlation between ROTEM parameters and areas of liver sinusoidal fibrin deposition. All data were reported as median and range. Differences were considered statistically significant at a *p* value of less than 0.05.

## 3. Results

### 3.1. LPS-Induced Coagulopathy and Liver Microthrombosis

**Figure 1:**
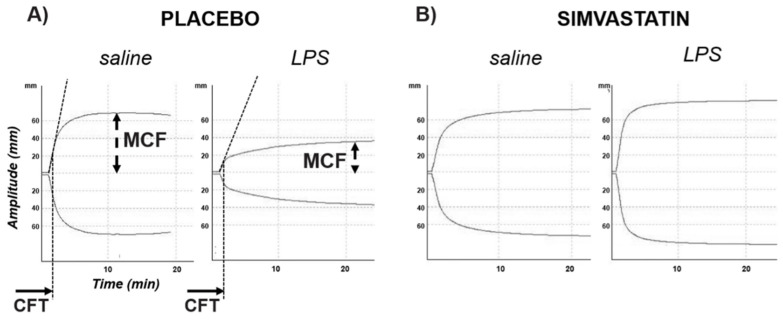
Representative ROTEM graphs showing the effect of LPS vs. saline on the in vitro clot formation in the placebo (**A**) and simvastatin treatment conditions (**B**). CFT, clotting formation time (sec); MCF, maximum clot firmness (units). More details on the curves are provided in Table 1.

**Figure 2:**
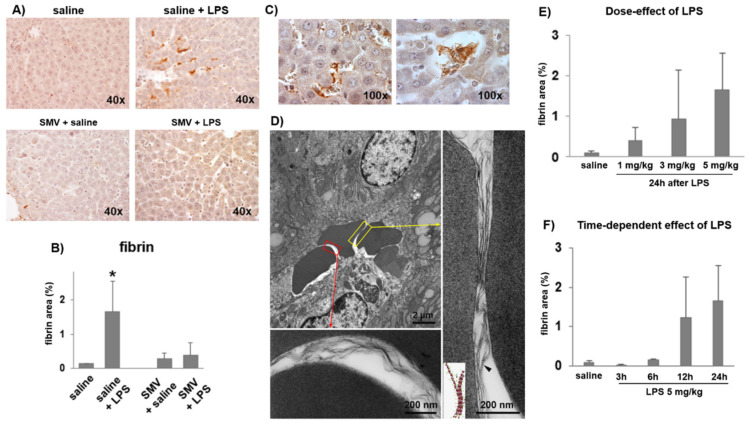
Fibrin deposition in the liver of rats 24 h after saline vs. LPS (5 mg/kg) in the placebo and simvastatin (SMV) group of treatment. (**A**) Fibrin was evident in liver sinusoids after LPS in the placebo treatment. (**B**) Bar-graphs showing fibrin content in liver sinusoids assessed by image analysis and expressed as %. Data are reported as mean ± SD. * *p* < 0.05 vs. saline. (**C**) Micrographs demonstrating fibrin deposition in a sinusoid (**left**) and in the central vein (**right**), showing that fibrin fibrils form a mesh entrapping erythrocytes. (**D**) Transmission electron micrographs of liver 24 h after LPS injection (5 mg/kg). Intrasinusoidal fibrin strands observed around some red blood cells in boxed areas are shown at higher magnification (red and yellow arrows). Arrowhead points to a fibrin strand exhibiting the typical period-based structure. The schematic representation of a fibrin strand is shown in the inset. (**E**) Bar-graphs showing fibrin deposition in rats 24 h after injection of increasing doses of LPS (1–3–5 mg/kg) or (**F**) different times after injection of LPS at 5 mg/kg (3–6 to 12–24 h). Data are reported as mean ± SD.

Figure 1 shows the most representative traces of clot generation by ROTEM on peripheral blood and Table 1 details the quantification of the variables describing the in vitro viscoelastometry. Twenty-four hours after injection, LPS was associated with a longer clotting formation time (CFT, sec) and a lower maximum clot firmness (MCF, mm) (*p* < 0.001) when compared to saline, thus defining the hypo-coagulability of peripheral blood. On the contrary, 24 h after LPS, livers had a significantly greater percentage of areas stained for fibrin than saline (*p* < 0.05) (Figure 2A,B), thus marking a hyper-coagulability at organ microcirculation. Fibrin deposition was also detectable inside larger intrahepatic blood vessels organized as fibrin-enriched thrombi embedded with red blood cells (Figure 2C). Electron microscopy analysis confirmed intrasinusoidal fibrin deposition showing sinusoids piled with red blood cells entrapped in an electron-dense material exhibiting the classic pattern of fibrin fibrils (Figure 2D). The greatest amount of fibrin deposition was found in liver specimens collected 24 h after LPS injection at 5 mg/kg in a dose- and time-dependent manner (Figure 2E,F, respectively). 

**Figure 3:**
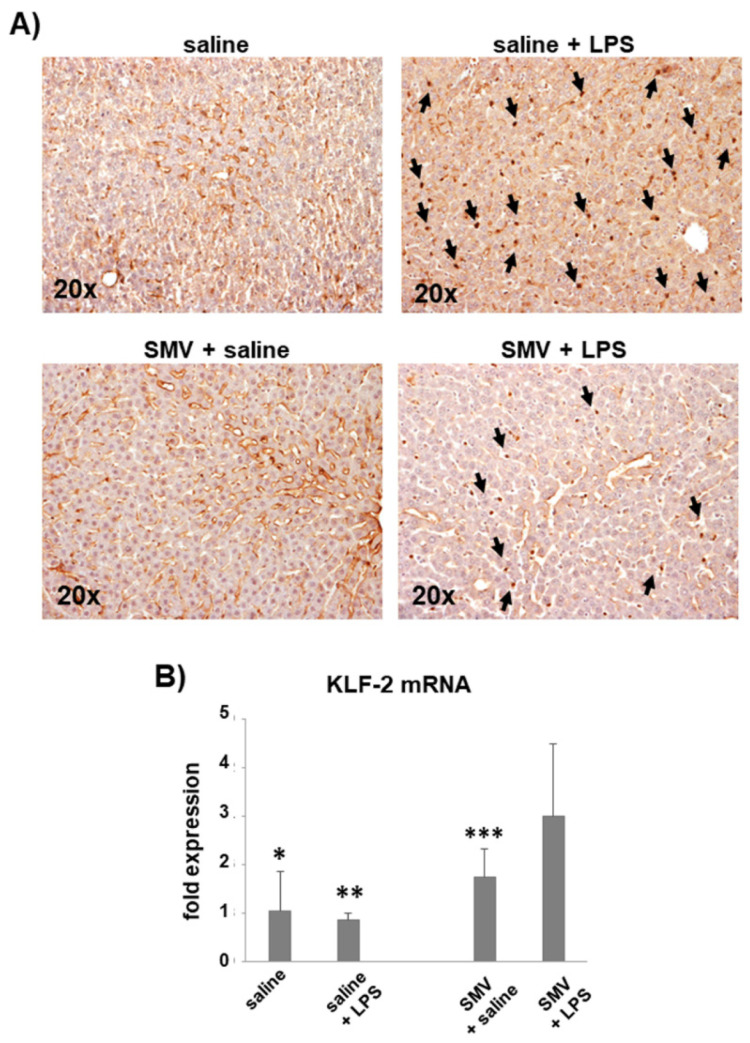
(**A**) TM expression in the liver of rats 24 h after saline vs. LPS (5 mg/kg) in the placebo and simvastatin (SMV) groups of treatment (immunohistochemistry). In the placebo group, immunoreactivity was detected in the endothelium lining the sinusoids after saline injection, while TM shedding was evident after LPS exposure (arrows). (**B**) Bar-graph showing KLF-2 gene expression. Data were normalized on 18 s mRNA levels and are reported as mean ± SD. * *p* < 0.01 vs. saline + LPS, SMV + saline and SMV + LPS; ** *p* < 0.01 vs. SMV + saline and SMV + LPS; *** *p* < 0.01 for SMV + saline vs. SMV + LPS.

**Figure 4:**
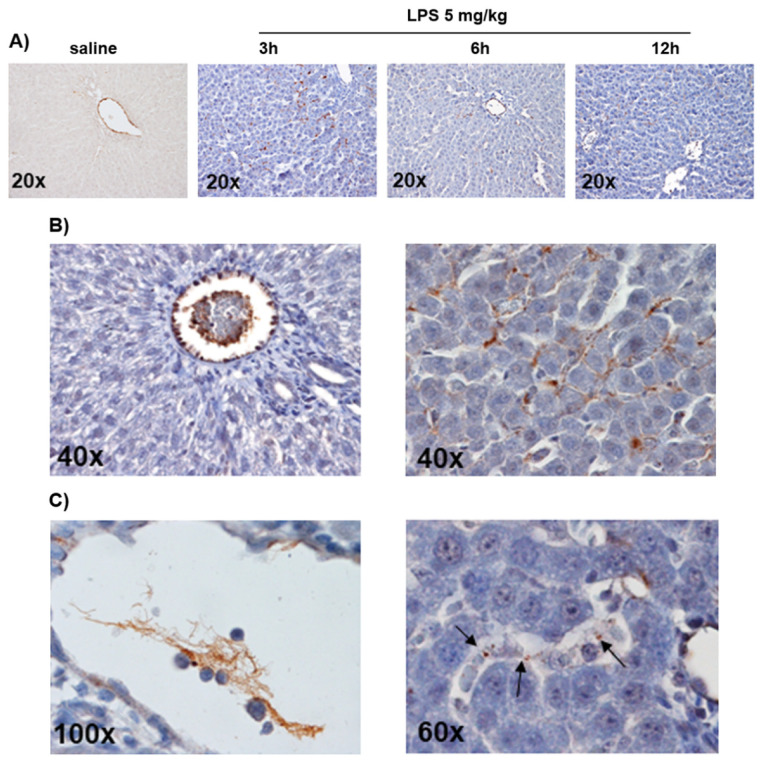
(**A**) VWF expression in the liver of rats 3–6–12 h after LPS (5 mg/kg) vs. saline injection (time 0). The expression of VWF shifted gradually from the endothelium of the portal tract at time 0, to inside sinusoids nearest the portal vessel, but outside endothelial cells, at time 3–6–12 h. (Original magnification: 20×) (**B**,**C**) VWF expression in liver sinusoids and large vessels 24 h after LPS injection (5 mg/kg) (immunohistochemistry). In panel (**B**), VWF was localized in endothelial cells of the portal tract and in thrombi inside the vessel (**left**) and was also detectable inside short tracts of sinusoids (**right**). In panel (**C**), VWF was evident in the mesh of thrombi inside blood vessels (**left**), as well as forming microthrombi inside sinusoids (arrows) (**right**).

Western blot analysis showed a similar expression of TM in liver samples from LPS- and saline-exposed rats (Supplementary Figure S2), however, immunohistochemistry revealed a different pattern of expression. Indeed, TM immunoreactivity was detectable in endothelial cells lining sinusoids in saline-injected rats while 24 h after LPS it was almost totally lost in endothelial cells but detectable as immunoreactive masses in nucleated cells engulfing the sinusoids (Figure 3A). We also found a gradient of VWF expression detected during the first 24 h after LPS injection. In detail, VWF was detectable at time 0 inside the endothelial cells of the portal tract (rats injected with saline), while, at time 3–6–12 h after LPS, its localization shifted gradually outside endothelial cells and inside the lumen of sinusoids closer to the portal vessels (Figure 4A). Finally, at 24 h after LPS, VWF was detected in endothelial cells of the portal tract and much less inside the short tracts of sinusoids, especially nearest the portal triad (Figure 4B). Meshes of fibrin-positive thrombi inside small and large intrahepatic vessels and microthrombi inside sinusoids were also positive for VWF (Figure 4C). The intensity of the VWF stain was apparently not influenced by higher doses of LPS (Supplementary Figure S3). 

### 3.2. Microthrombosis-Associated Liver Damage

**Figure 5:**
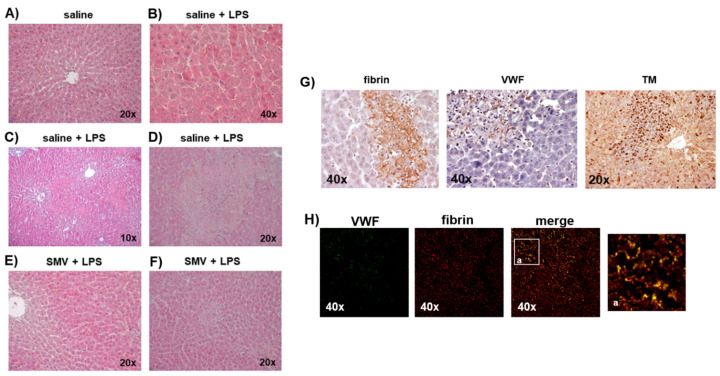
(**A**–**F**): Liver structure in the different groups of the main experimental protocol (hematoxylin and eosin). All liver samples were collected 24 h after saline vs. LPS-injection (5 mg/kg). (**G**,**H**): Immunohistochemistry for fibrin, VWF and TM in the liver of rats 24 h after LPS (5 mg/kg). (**A**) In saline rats, we observed a normal structure of the parenchyma. (**B**) In saline + LPS rats, an evident and diffuse eosinophilia was detected (**C**,**D**), as well as frequent areas of necrosis. (**E**) Simvastatin (SMV) was not able to protect from LPS-induced eosinophilia, (**F**) but liver necrosis was strongly reduced. (**G**) The expression of fibrin, VWF deposition and TM shedding were evident in necrotic areas of the liver parenchyma, suggesting their involvement in the liver injury induced by sinusoidal microthrombosis after LPS. (**H**) Immunofluorescence analysis of a clot from peripheral blood sample showing fibrin and VWF expression and co-localization (last image on the right side represents magnification of co-localization). Micrographs were obtained using a WD THUNDER Imager Tissue 3D. Green: VWF; red: fibrin.

**Figure 6:**
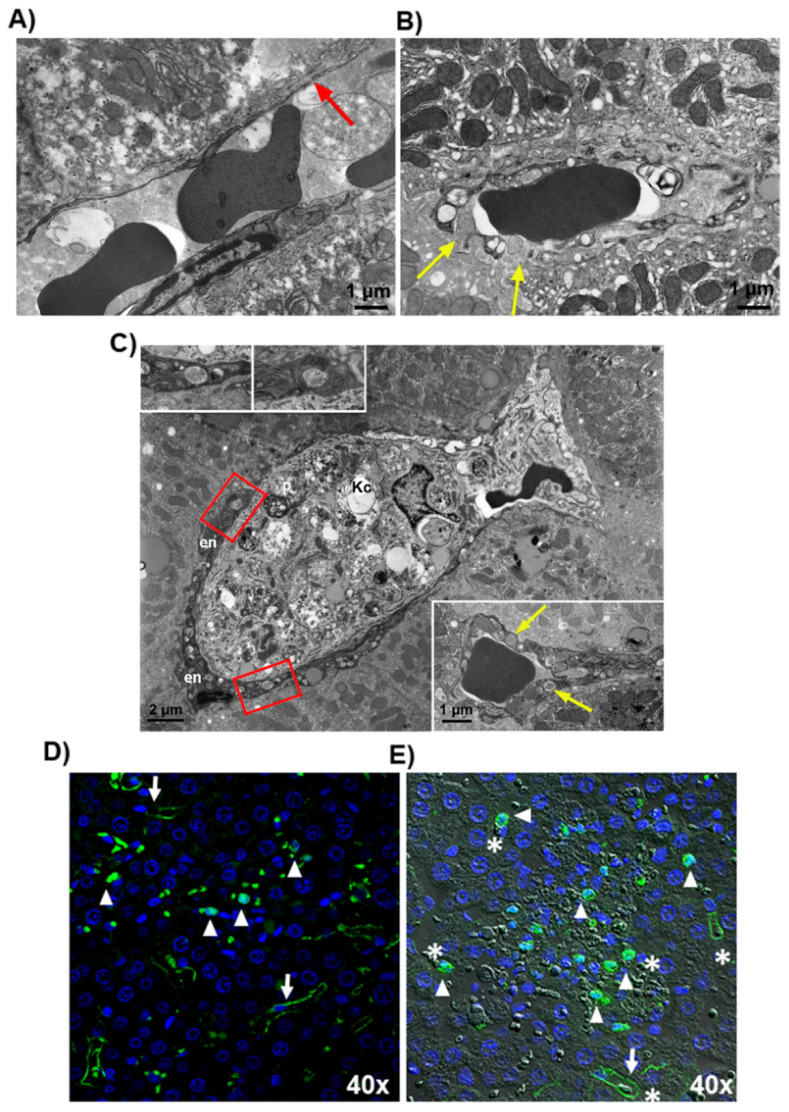
Representative rat liver from placebo group, saline-exposure: (**A**) a sinusoid with a physiologically discontinuous endothelium exhibiting a fenestration (red arrow) (transmission electron micrographs). Representative rat liver from placebo group, LPS exposure: (**B**) sinusoidal endothelium shows abnormally large fenestrations (yellow arrows) (transmission electron micrographs); (**C**) large Kupffer cell (Kc), completely occluding the sinusoid, that contains irregularly shaped phagosomes (*p*), red blood cell fragments and digestive debris. Note that endothelial cells (en) contain some electron-dense vacuoles, as shown in the boxed areas and enlarged in the insets in the upper part of the figure. Endothelial cells in the lower panel also exhibit electron-dense vacuoles (yellow arrows) (transmission electron micrographs); (**D**) TM was expressed in some endothelial cells lining liver sinusoids (arrows) or, more frequently, was shed forming rounded masses of the protein (arrowheads) (Immunofluorescence analysis); (**E**) localization of functional TM that lines sinusoids having erythrocytes inside (arrows), while TM shedding was detected in sinusoids where the endothelial line was lost (arrowheads) (differential interference contrast). The identity of sinusoids was confirmed by observing the position of erythrocytes (asterisks) that can be easily identified using this technique. The staining with DAPI revealed that the TM immunoreactive masses localized in the sinusoids exhibited the nucleus, suggesting they likely underwent phagocytosis by sinusoidal Kupffer cells. Green: TM; blue: DAPI.

Rats exposed to LPS showed more severe liver damage compared to those exposed to saline, as shown by a greater degree of eosinophilic and necrotic liver cells, which are markers of cellular stress and death, respectively. (Figure 5A–D). The histological grading results are summarized in Supplementary Table S4. Focal necrosis was more relevant in the periportal regions of LPS-exposed rats and associated with immunoreactivity for fibrin, VWF and TM (Figure 5G), thus confirming the involvement of the coagulation system and the endothelium in the pathogenesis of liver damage induced by endotoxemia. The colocalization of VWF with fibrin in clots used as positive controls was confirmed by the merged image (Figure 5H), in which VWF-positive fibrils were also fibrin-positive. Typically, inside the areas of liver damage, the endothelium showed signs of cellular stress such as dense granules inside the cytoplasm and abnormally large fenestrations consistent with the loss of endothelial cells and breaking of the endothelium lining the sinusoids, which were larger than expected (150–175 nm) based on physiologic fenestration (Figure 6A,B) [22]. Furthermore, Transmission Electron Microscopy revealed the presence of Kupffer cells in a tight relationship with injured endothelial cells in sinusoids (Figure 6C), suggesting a role for Kupffer cells in LPS-induced endothelial dysfunction. This observation was confirmed by immunofluorescence analysis. In fact, in those sinusoids where TM was still expressed by endothelial cells, the protein was detected as a continuous immunoreactive line (Figure 6D,E). In contrast, in those areas where immunoreactivity along sinusoids was lost, TM-positive aggregates were observed in DAPI stained cells, suggesting that they were Kupffer cells after the internalization of TM (see arrows and arrowheads in Figure 6D,E).

### 3.3. Simvastatin Effects on LPS-Induced Coagulopathy

The peripheral blood hypo-coagulability showed by ROTEM 24 h after exposure to LPS at 5 mg/kg was totally prevented by simvastatin (Figure 1B, Supplementary Table S4). In line with these data, simvastatin sensibly reduced the intrasinusoidal fibrin deposition associated with LPS (Figure 2A), and preserved sinusoidal TM expression, limiting its shedding from endothelial cells lining liver sinusoids in those rats exposed to LPS (Figure 3A). The levels of KLF-2 mRNA, the transcriptional factor of TM, were significantly up-regulated by simvastatin compared to the placebo regardless of LPS exposure (Figure 3B). In the histological sections of simvastatin treated rats, we observed a reduction of the number and size of necrotic areas following LPS exposure. Despite this, simvastatin could not prevent cellular stress as suggested by the persistence of a relatively high number of eosinophilic hepatocytes (Figure 5 and Supplementary Table S4).

**Figure 7:**
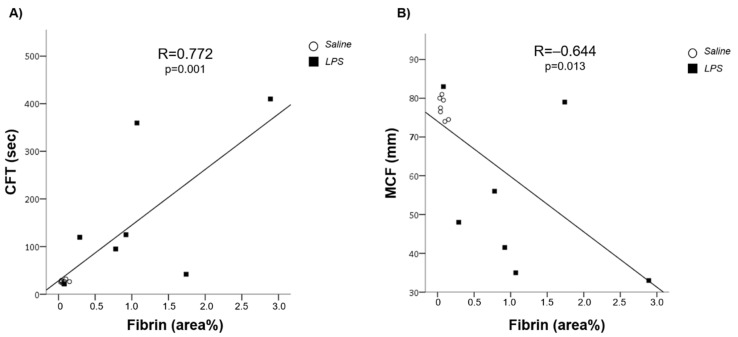
Correlation between fibrin clot formation time (**A**) and maximum clot firmness (**B**).

The hypo-coagulability of peripheral blood detected by ROTEM and expressed by the CFT and MCF correlated with the quantification of fibrin deposition (R = 0.772, *p* = 0.001; R = −0.644, *p* = 0.013, respectively) by confirming the association between the hypo-coagulability of peripheral blood with a condition of hyper-activation of the coagulation cascade at sinusoidal level (Figure 7).

## 4. Discussion

Sepsis is a common cause of in-hospital mortality as a consequence of intravascular thrombosis in the microcirculation, parenchymal necrosis and organ dysfunction. In this clinical setting, the onset of coagulopathy due to organ microthrombosis confers a mortality risk that progressively increases up to 56% [6]. In the present study, in a rat model of endotoxemia, the pharmacological modulation of endothelial cell function with simvastatin was associated with a significant antithrombotic effect on liver microcirculation after exposure to LPS. This effect on organ microcirculation correlated with the amelioration of the peripheral hypo-coagulability assessed by ROTEM, a global assay on whole blood. All these findings are in line with the hypothesis that the protection of the endothelium may prevent organ microthrombosis and sepsis-associated coagulopathy. Moreover, results are coherent with previous observation on ROTEM profiles in animal models of LPS-induced hemostasis activation [23,24,25] and expand to previous observations on liver hemodynamic response to LPS [10,12]. Interestingly, ROTEM hypo-coagulable features resemble those changes already described in patients with decompensated cirrhosis and acute chronic liver failure, which are clinical conditions characterized by an inflammatory milieu due to circulating bacterial products [26]. 

Blood coagulation is a tightly regulated enzymatic system and vascular endothelial cells are of utmost importance to preserve organs from microthrombosis [27]. In this study, we considered TM expression as a marker of healthy endothelium in liver microcirculation because this transmembrane protein, in physiologic conditions, contributes to the anticoagulant activity of endothelial cells. Beyond this role as a biomarker of a healthy endothelium, some authors have considered a proper cause–effect relation of TM with organ microthrombosis by ultimately proposing TM as a target of therapy to prevent or treat sepsis-associated coagulopathy [28,29]. As matter of fact, Kume et al. demonstrated that LPS caused intrasinusoidal fibrin deposition in the liver in association with loss of TM expression in sinusoidal endothelial cells, whereas fibrin deposition was totally prevented by the infusion of soluble TM [17]. Unfortunately, the benefits of interventions providing direct inhibition of the coagulation system, such as TM infusion or other anticoagulant strategies, although clear in animal models, have not been firmly confirmed in humans [30,31]. Phase II and III clinical trials, in fact, suggested that anticoagulants did provide survival benefits in highly selected patients, if, however, at the expense of major hemorrhagic complications [30]. The present study indirectly confirms the role of TM to preserve the liver from microthrombosis during endotoxemia, but, at variance from previous experiences, the antithrombotic effect was achieved by simvastatin, which preserved the endothelial expression of TM and did not directly inhibit coagulant factors. This mechanism could translate into several clinical advantages. Firstly, anticoagulation promoted by this approach is rather physiologic as it does not directly target coagulation factors, thereby not interfering with the tight feedback control systems of hemostasis. Hence, the potential hemorrhagic risk of this anticoagulant strategy may be minimal, although this advantage should be confirmed by additional studies on bleeding models. Secondly, in our hands, simvastatin induced TM expression in a site-specific manner targeting dysfunctional endothelial cells, as shown by the loss of TM expression in sinusoidal endothelial cells caused by LPS that was blunted by simvastatin, coherently with an endothelial-specific action of the drug. Lastly, statins have already been suggested as a potential additional therapy for the management of sepsis [32] and a metanalysis showed that patients receiving statins for the management of cardiovascular risk and dyslipidemia may have a survival advantage during sepsis [33]. These latter findings could be translated into a premise for new randomized controlled trials in humans, which could also definitively fill the gap of knowledge on the safety profile of statins in this clinical setting, as already explored in human studies, especially in conjunction with coenzyme Q administration [34,35,36]. Along these lines, our data demonstrate that simvastatin prevented LPS-induced microthrombosis, which translated into a reduced number of foci of necrosis detected in the liver. These results are coherent with seminal papers published during the last 10 years and systematically describe a hepatoprotective effect of simvastatin and other biosimilars, to the point of considering statins a potential class of drugs to prevent liver-related mortality in daily practice [16]. This important achievement is sustained by the so-called pleiotropic effects of statins predominantly based on the inhibition of isoprenoid-synthesis [37,38] the biochemical pathway modulating post-translational factors via isoprenylation. The interference of statins with this cellular system of control causes up-/down-regulation of several enzymes participating in inflammation, angiogenesis, redox balance and vascular physiology. In this study, we paid attention to the ability of simvastatin to preserve the expression of TM in sinusoidal endothelial cells via the induction of its transcriptional factor KLF-2, which was associated with the protection of the liver from microthrombosis. Importantly, KLF-2 is also a positive transcriptional factor of eNOS, the enzyme which enhances hepatic resistance to portal blood flow through NO-release from sinusoidal endothelial cells. Therefore, it is biologically plausible that both effects on the endothelium can act synergistically to preserve liver parenchyma during sepsis [10,12,26,39]. 

We acknowledge that this study suffers from some limitations. First, the experimental design, which entails the administration of simvastatin prior to LPS exposure, does not properly reflect the clinical scenario. Notwithstanding, as already demonstrated, simvastatin preserves sinusoidal endothelial cell function during endotoxemia in both preventive and acute schemes of administration [10]. Therefore, the main finding of our study consists of demonstrating that simvastatin interferes with the so-called sepsis-associated coagulopathy. Of course, this pharmacological effect should be investigated in clinical studies to draw a definitive therapeutic indication in humans. Second, no evaluation of platelets nor fibrinolysis was performed even though they deserve a role in endotoxemia and acute liver injury [40,41]. Unfortunately, the amount of blood available from each experiment did not allow us to offer an exhaustive overview of all the mechanisms triggering fibrin deposition into the sinusoids and systemic changes of hemostasis. However, we found a significant correlation between fibrin deposition into liver sinusoids, a marker of an organ hyper-coagulable state, and ROTEM data from peripheral blood, showing an in vitro hypo-coagulable profile by this global hemostatic test [42]. This correlation is in line with a consumption coagulopathy frequently observed in patients with disseminated intravascular coagulation, and the roles of the coagulation cascade, platelets and fibrinolysis have been already addressed by several authors [7,43].

Furthermore, as statins possess several anti-hemostatic properties other than preserving TM expression in endothelial cells, the pharmacological pathways modulated by simvastatin in our research are only partially described. Indeed, statins can interfere with primary hemostasis, secondary hemostasis and fibrinolysis [44,45]. Among them, the most interesting relative to our model is the inhibition of platelet activation, which can be another downstream effect of NO increase, the reduction of tissue factor expression as a consequence of KLF-2 increase, the pro-fibrinolytic activity as a consequence of the down-regulation and the up-regulation of plasminogen activator inhibitor-1 and tissue-type plasminogen activator, respectively. These mechanisms protect vessels from occlusion by abnormal fibrin-rich thrombi. Accordingly, it can be surmised that they act together with the induction of sinusoidal TM expression to prevent liver microthrombosis. Another limitation is that we do not know whether the observed pharmacologic effects of simvastatin apply to other statins. This is an important point since organ dysfunction during sepsis can influence the efficacy and toxicity of drugs and different types of statins could have different pharmacokinetic profiles with an important impact on their applicability. A recent in vitro study with sinusoidal endothelial cells showed several statins to share the ability to increase KLF-2 expression, yet simvastatin showed higher efficacy, possibly as a consequence of its lipophilic profile, which makes it more efficient to cross plasma membranes of endothelial cells with [11]. We also acknowledge that statins may be effective on microcirculation through the modulation of inflammation. This is not a trivial point considering that inflammation and endothelial dysfunction can synergistically enhance thrombosis [46] and TM itself can have a modulatory role on inflammation beyond hemostasis [29]. Although inflammation was not the target of our study, we surmise that statins could also influence this endothelium-dependent mechanism of disease. 

## 5. Conclusions

In conclusion, this study associates LPS with the development of intravascular thrombosis causing liver damage and demonstrates that simvastatin represents a potential endothelium-targeted strategy to preserve liver microcirculation and hemostasis during endotoxemia. Clinical studies are warranted to translate these pre-clinical results into protocols of treatment for sepsis in humans.

## Figures and Tables

**Table 1:** Rotational thromboelastometry (ROTEM©) data on whole blood samples. Comparison of lipopolysaccharide (LPS) vs. saline in placebo and simvastatin (SMV) experimental condition. Results are presented as median (min–max).

	PLACEBO			SIMVASTATIN		
	Saline	LPS 5 mg/kg	*p*	Saline	LPS 5 mg/kg	*p*
**CT** (**sec**)	53 (41–62)	67 (51–94)	0.126	53 (42–65)	50 (46–66)	1.000
**CFT** (**min**)	27 (23–33)	146 (42–410)	0.004	27 (26–32)	95 (22–120)	0.700
**MCF** (**mm**)	77 (70–81)	40 (33–79)	0.030	78 (74–80)	56 (48–83)	0.700
**MCF-t** (**sec**)	1742 (578–1891)	3336 (2095–3460	0.004	1581 (1533–2008)	2720 (1267–3096)	0.700
**Alfa-angle** (**°**)	85 (84–86)	77 (70–82)	0.004	85 (84–85)	80 (79–86)	0.700
**Max-V** (**velocity**)	46 (45–59)	19 (12–34)	0.004	50 (40–53)	23 (22–58)	0.700
**AUC**	7574 (6935–8048)	4023 (3305–7906)	0.052	7659 (7292–7905)	6415 (4691–8139)	0.700

## Data Availability

Not applicable.

## Supplementary Materials

The following supporting information can be downloaded at https://www.mdpi.com/article/10.3390/cells11071148/s1: Figure S1, Schematic representation of experimental groups; Figure S2, Representative Western blot analysis showing TM expression in liver homogenates in the main experimental protocol and Bar graphs showing TM expression after densitometric analysis of immunoreactive bands; Figure S3, Representative micrographs of immunohistochemistry analysis showing VWF expression in the liver of rats after 24 h from injection of 1–3–5 mg/kg of lipopolysaccharide (LPS); Table S1, morphometry of rats—Comparison of saline lipopolysaccharide (LPS) in placebo and simvastatin (SMV) experimental condition; Table S2, morphometry of rats in the experimental groups participating to the analysis of the lipopolysaccharide (LPS) dose-effect response; Table S3, morphometry of rats in the experimental groups participating to the analysis of the lipopolysaccharide (LPS) effects at several time point after injection; Table S4, Histological grading for the analysis of liver sections stained with haematoxylin and eosin in the main experimental protocol. Antibodies and DNA/cDNA Clones and Uncropped Western Blot Images can be found in Supplementary Materials.

## Abbreviations

CT, clotting time; CFT, clot formation time; DAPI, 4′,6-diamidino-2-phenylindole; eNOS, endothelial nitric oxide synthase; KLF-2, Kruppel-like-factor-2; LPS, lipopolysaccharide; MCF, maximum clot firmness; MCF-T, maximum clot firmness time; TM, Thrombomodulin; ROTEM, rotational thromboelastometry; VWF, Von Willebrand Factor.
