# Supplementary material for: Simvastatin Prevents Liver Microthrombosis and Sepsis Induced Coagulopathy in a Rat Model of Endotoxemia
Source: Cells. 2022 Mar 29;11(7):1148. doi: 10.3390/cells11071148 (PMC8997834; doi:10.3390/cells11071148)
Supplement: Supplementary file 1 [file cells-11-01148-s001.zip › cells-1582415-supplementary.pdf]

Simvastatin preserves the antithrombotic environment of the liver and prevents sepsis-associated coagulopathy in a rat model of endotoxemia

Vincenzo La Mura [1,2,3], Nicoletta Gagliano[3], Francesca Arnaboldi[3], Patrizia Sartori[3], Patrizia Procacci[3], Luca Denti[3], Eleonora Liguori[3], Niccolò Bitto[1], Giuseppe Ristagno[4,5], Roberto Latini[4], Daniele Dondossola[3,6], Francesco Salerno[3], Armando Tripodi[1], Massimo Colombo[7], Flora Peyvandi[1,5].

Supplementary materials and methods: table of contents

Supplementary Figure S1 2

Supplementary Figure S2 3

Supplementary Figure S3 3

Supplementary Table S1 4

Supplementary Table S2 4

Supplementary Table S3 5

Supplementary Table S4 5

Supplementary-Uncropped Western Blot Images 6

Supplementary-Antibodies and DNA/cDNA Clones 7

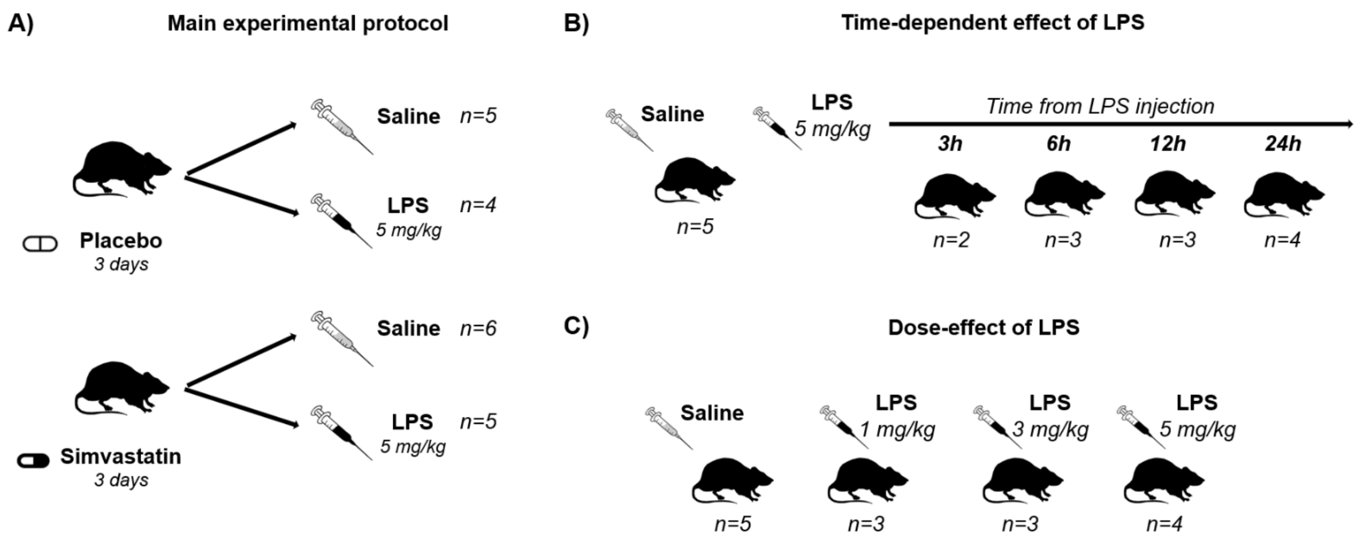

Supplementary-Figure S1. Schematic representation of experimental groups: A) Main experimental protocol: placebo and 3-day pre-treatment with simvastatin in rats exposed to LPS (5mg/Kg) or saline (24 hours from intraperitoneal injection), B) time-dependent effect protocol, C) dose-effect protocol.

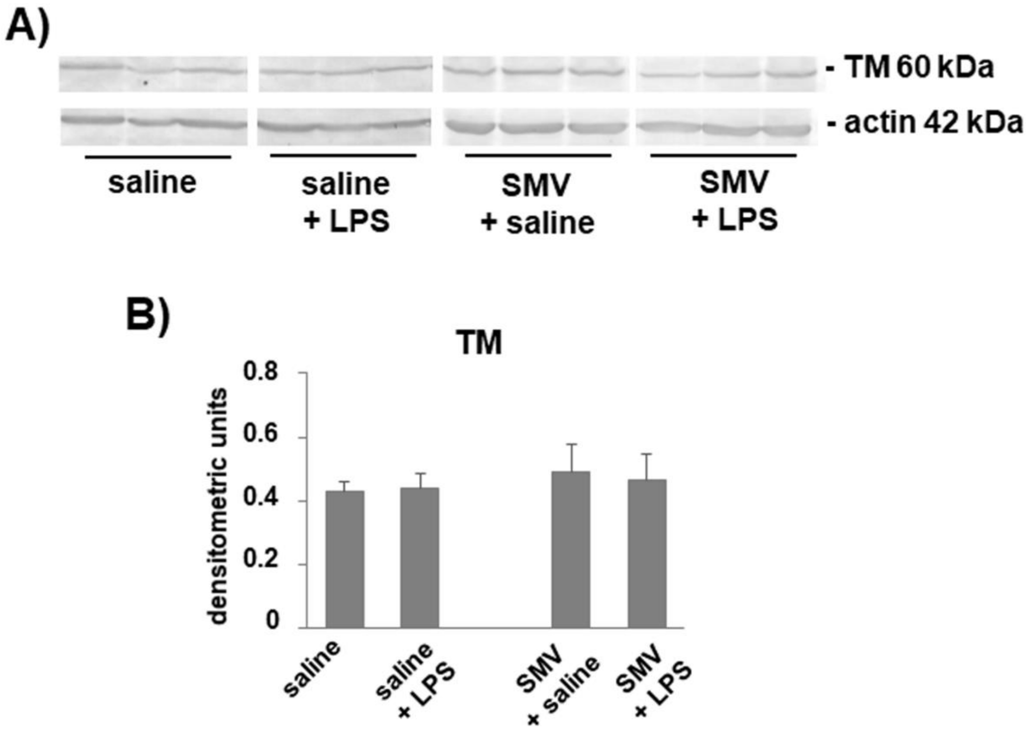

Supplementary-Figure S2. A) Representative Western blot analysis showing TM expression in liver homogenates in the main experimental protocol. Data were normalized on actin expression. (SMV=Simvastatin; LPS=Lipopolysaccharide) **B**) Bar graphs showing TM expression after densitometric analysis of immunoreactive bands. Data are reported as mean ± SD.

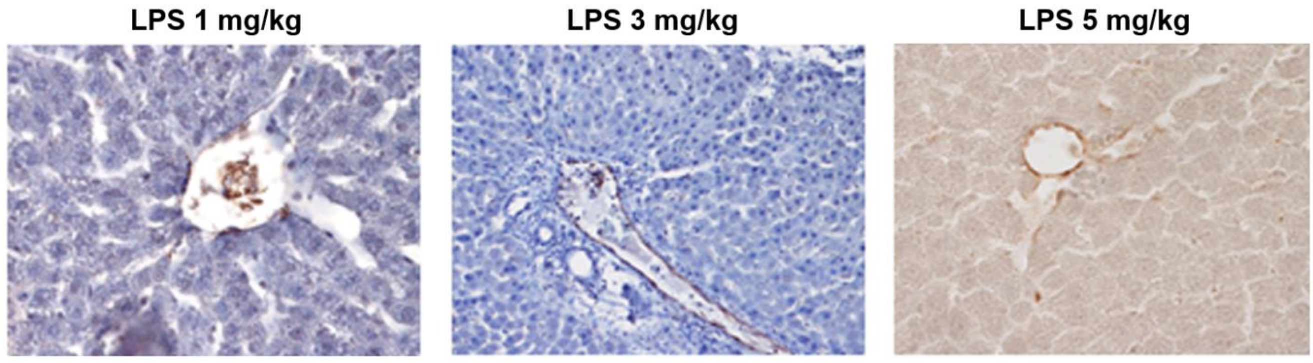

Supplementary-Figure S3. Representative micrographs of immunohistochemistry analysis showing VWF expression in the liver of rats after 24 hours from injection of 1-3-5 mg/Kg of lipopolysaccharide (LPS). Increasing doses of LPS did not apparently influence VWF expression intensity and localization. (Original magnification: 20x)

Supplementary-Table S1: morphometry of rats. Comparison of saline lipopolysaccharide (LPS) in placebo and simvastatin (SMV) experimental condition

	**PLACEBO**			**SMV**		
	**saline**	**LPS 5mg/Kg**	P	**saline**	**LPS 5 mg/Kg**	P
**Number**	*5*	*4*		*6*	*5*	
**Body weight** (gr)	313 (306-352)	346 (325-407)	0.063	335 (310-371)	326 (315-333)	0.662
**Liver weight** (gr)	15 (14-18)	12 (11-15)	0.111	16 (11-19)	12 (11-16)	0.095
**Spleen weight** (gr)	1.5 (1.1-1.7)	0.9 (0.8-1.3)	0.063	1.1 (0.8-1.5)	1.0 (0.8-1.0)	0.690

Results are presented as median(min-max)

Supplementary-Table S2: morphometry of rats in the experimental groups participating to the analysis of the lipopolysaccharide (LPS) dose-effect response

	**Saline**	**LPS 1 mg/Kg**	**LPS 3 mg/Kg**	**LPS 5 mg/Kg**	P
**Number**	*5*	*3*	*3*	*4*	
**Body weight** (gr)	313 (306-352)	288 (275-312)	277 (275-312)	346 (325-407)	0.379
**Liver weight** (gr)	15 (14-18)	14 (12-15)	12 (11-12)	12 (11-15)	0.014
**Spleen weight** (gr)	1.5 (1.1-1.7)	1.0 (0.9-1.0	1.1 (1.1-1.1)	0.94 (0.8-1.3)	0.034

Results are presented as median(min-max)

Supplementary-Table S3: morphometry of rats in the experimental groups participating to the analysis of the lipopolysaccharide (LPS) effects at several time point after injection

	**saline**	**LPS 5 mg/Kg 3h**	**LPS 5 mg/Kg 6h**	**LPS 5 mg/Kg 12h**	**LPS 5 mg/Kg 24h**	P
**number**	*5*	*2*	*3*	*3*	*4*	
**Body weight** (gr)	313 (306-352)	323 (318-328)	320 (320-320)	330 (306-333)	346 (325-407)	0.018
**Liver weight** (gr)	15 (14-18)	15 (14-16)	13 (12-13)	12 (11-14)	12 (11-15)	0.061
**Spleen weight** (gr)	1.5 (1.1-1.7)	1.1 (1.1-1.1)	1.2 (0.9-1.3)	1.0 (0.8-1.1)	0.94 (0.8-1.3)	0.100

Results are presented as median(min-max)

Supplementary-Table S4: Histological grading for the analysis of liver sections stained with haematoxylin and eosin in the main experimental protocol. (SMV=Simvastatin; LPS=Lipopolysaccharide) Hepatocyte eosinophilia and liver necrosis were graded as follows: - absence, + mild, ++ moderate, +++ severe.

	**necrosis**	**eosinophilia**
**saline**	-	+/-
**saline + LPS**	++/+++	++
**SMV + saline**	-	+/-
**SMV + LPS**	+	+/++

Supplementary-Uncropped Western Blot Images

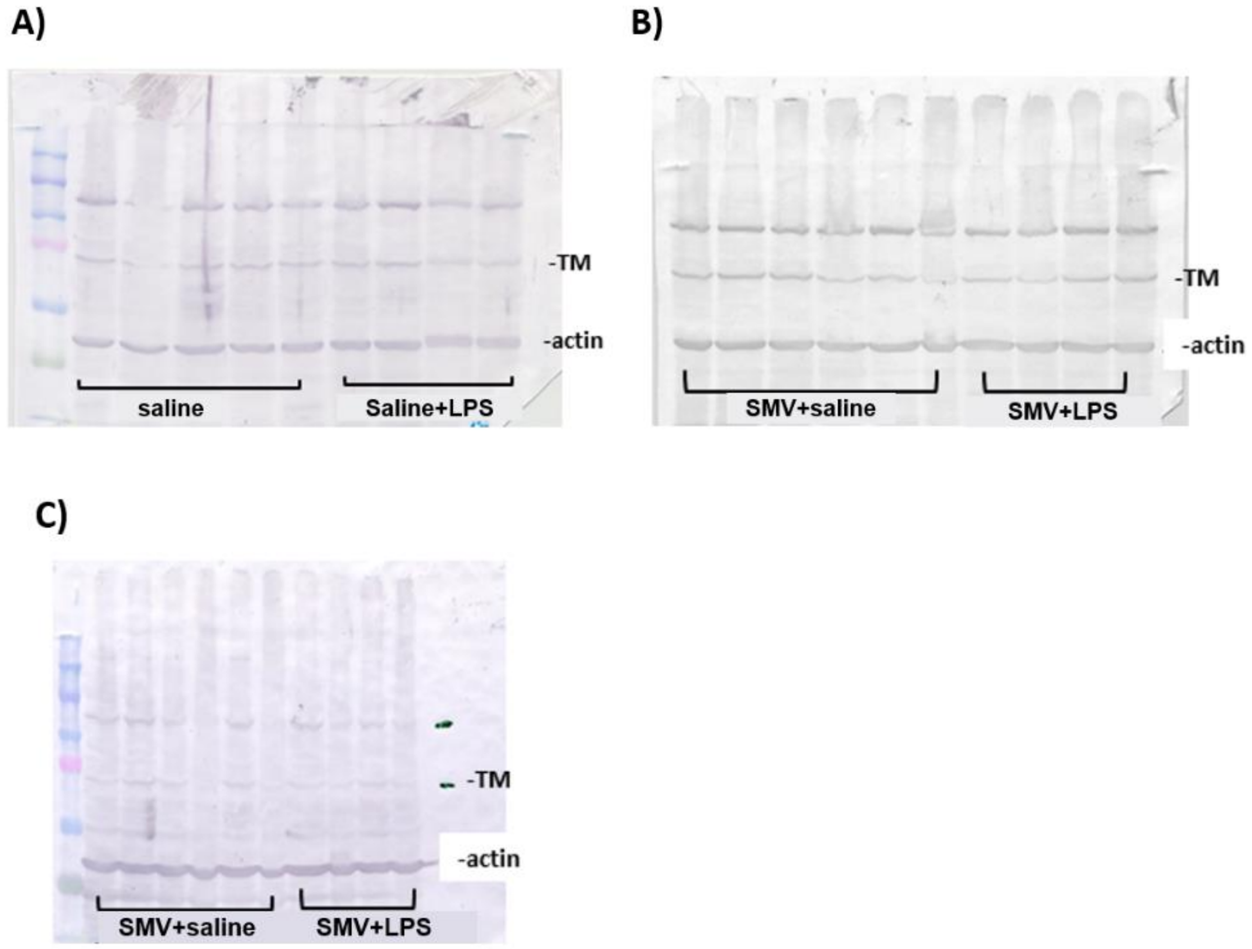

Uncropped Western Blot Images of supplementary Figure S2, showing TM expression in liver homogenates in the main experimental protocol. (SMV=Simvastatin; LPS=Lipopolysaccharide).

Supplementary-Antibodies and DNA/cDNA Clones

Antibodies

**Target antigen**	**Vendor or Source**	**Catalog #**	**Working concentration**	**Lot # (preferred but not required)**	**Persistent ID / URL**
**Primary antibodies**					
goat anti-fibrinogen GAHu/Fbg/7S	Nordic-MUbio	GAHu/Fbg/7S	dilution 1:400 in PBST		
sheep anti-Thrombomodulin (TM)	R&DSystems	AF3947	1:20 in PBST		
rabbit monoclonal anti-von Willebrand factor (VWF) (,)	Cell Signaling	65707	1:500 in PBST	D8L8G	
mouse monoclonal anti-β-actin	Sigma-Aldrich	A5441	1:7500 in TBST	AC-15	
**Secondary antibodies**					
rabbit anti-goat HRP conjugated	Thermo Scientific	81-1620	dilution 1:400 in PBST		
rabbit anti-sheep HRP conjugated	Novus	NB7195	1:6000 in TBST		

goat anti-rabbit HRP conjugated	Novus	NB7160	1:6000 in TBST		
anti-mouse HRP conjugated	Sigma-Aldrich	A9044	1:6000 in TBST		
goat anti-rabbit Alexa488 conjugated	Jakson ImmunoResearch	111-545-045	1:500 in PBST		

DNA/cDNA Clones

**Clone Name**	**Sequence**	**Source / Repository**	**Persistent ID / URL**
KLF-2 sense	ACTTGCAGCTACACCAACTG	IDT Integrated DNA Technologies, Belgium	
KLF-2 antisense	CTGTGACCCGTGTGCTTG	IDT Integrated DNA Technologies, Belgium	
18s sense	CTGCCCTATCAACTTTCGATGGTAG	IDT Integrated DNA Technologies, Belgium	
18s antisense	CCGTTTCTCAGGCTCCCTCTC	IDT Integrated DNA Technologies, Belgium	
